# Making the Genotypic Variation Visible: Hyperspectral Phenotyping in Scots Pine Seedlings

**DOI:** 10.34133/plantphenomics.0111

**Published:** 2023-11-14

**Authors:** Jan Stejskal, Jaroslav Čepl, Eva Neuwirthová, Olusegun Olaitan Akinyemi, Jiří Chuchlík, Daniel Provazník, Markku Keinänen, Petya Campbell, Jana Albrechtová, Milan Lstibůrek, Zuzana Lhotáková

**Affiliations:** ^1^ Department of Genetics and Physiology of Forest Trees, Faculty of Forestry and Wood Sciences, Czech University of Life Sciences Prague, Prague, Czech Republic.; ^2^ Department of Environmental and Biological Sciences, University of Eastern Finland, Joensuu, Finland.; ^3^ Department of Experimental Plant Biology, Charles University, Prague, Czech Republic.; ^4^ Center for Photonic Sciences, University of Eastern Finland, Joensuu, Finland.; ^5^ Department of Geography and Environmental Sciences, University of Maryland Baltimore County, Baltimore, MD, USA.; ^6^ Biospheric Sciences Laboratory, NASA Goddard Space Flight Center, Greenbelt, MD, USA.

## Abstract

Hyperspectral reflectance contains valuable information about leaf functional traits, which can indicate a plant’s physiological status. Therefore, using hyperspectral reflectance for high-throughput phenotyping of foliar traits could be a powerful tool for tree breeders and nursery practitioners to distinguish and select seedlings with desired adaptation potential to local environments. We evaluated the use of 2 nondestructive methods (i.e., leaf and proximal/canopy) measuring hyperspectral reflectance in the 350- to 2,500-nm range for phenotyping on 1,788 individual Scots pine seedlings belonging to lowland and upland ecotypes of 3 different local populations from the Czech Republic. Leaf-level measurements were collected using a spectroradiometer and a contact probe with an internal light source to measure the biconical reflectance factor of a sample of needles placed on a black background in the contact probe field of view. The proximal canopy measurements were collected under natural solar light, using the same spectroradiometer with fiber optical cable to collect data on individual seedlings’ hemispherical conical reflectance factor. The latter method was highly susceptible to changes in incoming radiation. Both spectral datasets showed statistically significant differences among Scots pine populations in the whole spectral range. Moreover, using random forest and support vector machine learning algorithms, the proximal data obtained from the top of the seedlings offered up to 83% accuracy in predicting 3 different Scots pine populations. We conclude that both approaches are viable for hyperspectral phenotyping to disentangle the phenotypic and the underlying genetic variation within Scots pine seedlings.

## Introduction

Changes under global climatic conditions necessitate the selection of more resistant tree genotypes in forestry [1,2]. Exploring intrapopulation variability may be the crucial first step in curating adaptations to the future climate in tree species with long-generation cycles. Scots pine has one of the largest distribution ranges and grows naturally across various climatic conditions, creating different ecotypes [3,4]. Some ecotypes may be more suitable for afforestation of specific sites due to their resistance to various environmental stresses [5,6]. Selection of resistant individuals based on their phenotype may not be reliable [7] because of environmental effect; instead, selection must be based on genetic estimates after a proper design.

High-throughput phenotyping (HTP) has become widely utilized in crop science [8,9]; however, in forestry, it still needs more research and verification to be adopted [10,11]. The use of HTP is driven by the need to understand plant performance and growth response to environmental stresses and pathological agents and to offer solutions to various breeding scenarios [8,12]. Managing large-scale phenotypic data remains one of the major bottlenecks to plant breeding and functional genomics [11,13].

Vegetation 350- to 2,500-nm [visible to shortwave infrared (VSWIR)] reflectance data contain valuable information about the leaf’s chemical composition, such as photosynthetic pigments [14–16], water content [17,18], and structural compounds [19–22] that correspond to plant physiological status [23–26], and growth parameters [27] changing with leaf phenology during the vegetative season [28,29]. Therefore, leaf functional traits derived from seedlings’ hyperspectral reflectance are potentially a powerful tool in tree nurseries for evaluating the quality of the seedlings. We suppose that some leaf functional traits influencing optical response are provenance-specific; thus, if the seedling provenance is traceable through VSWIR reflectance data, hyperspectral phenotyping can serve to avoid possible maladaptation of seed sources. Even in certified stands where reproduction material is collected, there are still uncertainties about the actual origin and the true ecotypic features due to many decades of artificial regeneration [30,31]. Another benefit of the leaf functional traits detected using hyperspectral reflectance could be the predictions of the seedling’s performance under stress, which could help identify seedlings unsuitable for deployment. Using optical leaf data, it is possible to monitor seedling infections already at the nursery [32].

Since the early 1990s, accurate laboratory and field measurements of hyperspectral reflectance have been made with spectroradiometers covering spectra from the visible (VIS) through the near-infrared (NIR) to the SWIR [33]. The resulting reflectance captures the information about the leaf or canopy properties: The reflectance in the VIS part of the spectrum (300 to 700 nm) responds primarily to pigment content [34]; NIR (750 to 1,350 nm) reflectance is influenced by the internal structure of the leaves [35] or canopy structure [36] and water content [37]. The sharp increase in reflectance between VIS and NIR is called the “red edge” (RE), is usually defined by a wavelength range of 680 to 750 nm [38], and is known to be indicative of nitrogen levels and stress, e.g., [24,25,39]. SWIR spectral region could be subdivided into SWIR1 (1,350 to 1,800 nm) and SWIR2 (2,000 to 2,400 nm), and, here, the reflectance is dependent on the content of water and structural compounds of the leaves [40–42].

Multispectral [43] or hyperspectral optical sensors [44] could detect the leaf optical properties and chlorophyll content at the canopy level. This approach is usually applied in remote sensing studies using satellite, airborne, or unmanned automated vehicle sensors. Although hyperspectral cameras with spatial resolution can be used under laboratory conditions, spectroradiometers without spatial resolution are still viable for detecting leaf physiological properties without labor-intensive image segmentation [45], a primary bottleneck in image-based spectrometry. These spectroradiometers can be used under the field or laboratory conditions, using a contact or noncontact approach. Commonly used field spectroradiometers are equipped with a fiber optical cable (OC) with optional attachable equipment, such as a contact probe (CP) with an internal light source [21,42]. Each measurement approach has a different light source and sensor geometry, providing specific advantages regarding speed, laboriousness, and field operability. The reflectance data acquired by these measurement approaches are not fully compatible, and, therefore, they are often compared with each other: OC versus CP [37,46,47].

The CP is designed to measure the reflectance of planar leaves (at the leaf level) that optimally cover its entire field of view (1 cm^2^). In planar leaves, which evenly cover the field of view, CP measurements are simple and fast. However, in the case of conifers, issues such as needle overlapping and gap fraction between the needles need to be addressed for contact measuring, making it a challenging and time-consuming process [48]. Nevertheless, some studies have measured the needle optical properties by CP on shoot samples [49], stacked in one layer [46,50], or fixed in a custom-made holder for integration sphere [48,51,52].

A spectroradiometer outfitted with a CP attached to the OC allows us to obtain a biconical reflectance factor (BCRF) [53]. Using the OC to measure the object and a white panel at a distance under an external light source (i.e., solar light or an artificial one), we obtain a hemispherical conical reflectance factor (HCRF). BCRF focuses on a limited range of incident light within a conical solid angle, while HCRF considers light coming from the entire hemisphere, in our case, ambient sky illumination [53,54]. Both reflectance quantities BCRF and HCRF are expressed as relative values related to the reflectance of the white reference panel measured in a given setup.

The leaf’s internal structure affects its optical properties measured by a spectroradiometer equipped with the CP [55] or the integration sphere [56]. However, twig, branch, or canopy morphology, which could be ecotype specific, is not influencing the CP spectral signal. The OC alone can be used for proximal/canopy hyperspectral reflectance measurements to yield reproducible results, albeit highly susceptible to ambient light and atmospheric conditions [54]. The main advantages of the OC method under stable light conditions are its relatively higher speed in contrast to the CP method and the larger field of view, depending on the proximity to the measured object.

Moreover, the OC reflectance acquisition enables canopy level measurement of small plants. Thus, the spectral information also involves signals from the canopy structure and other morphological features of the seedlings unattainable by the CP measurements. The prediction and classification of samples using machine learning (ML) algorithms with VSWIR reflectance are known to the scientific community, including various applications in forestry. It is important to note that the reflectance data have been used to classify trees accurately at the species level [57,58]. Recently, emerging studies successfully used reflectance spectra to classify plant groups at the species level, i.e., interspecific hybrids [59,60] and local populations of single species [42]. Nevertheless, a comparative case study in BCRF and HCRF in an ML context to detect various conifer seedling populations/ecotypes is missing.

In the present study, we compare the potential of using the OC versus CP for the fast and efficient manual HTP of Scots pine seedlings originating from 3 local populations corresponding to 3 seed orchards. The specific aims were to (a) demonstrate that both approaches are viable for disentangling the phenotypic and potential genetic variation within the 3 populations of Scots pine seedlings; (b) evaluate which spectral regions (from VIS to SWIR) were the most sensitive in population distinction; (c) predict the given origin of seedlings by 2 selected ML algorithms -[random forest (RF) and support vector machine (SVM)] based on reflectance factor acquired by CP (BCRF) and OC (HCRF).

## Materials and Methods

### Study site and plant material

**Fig. 1.**
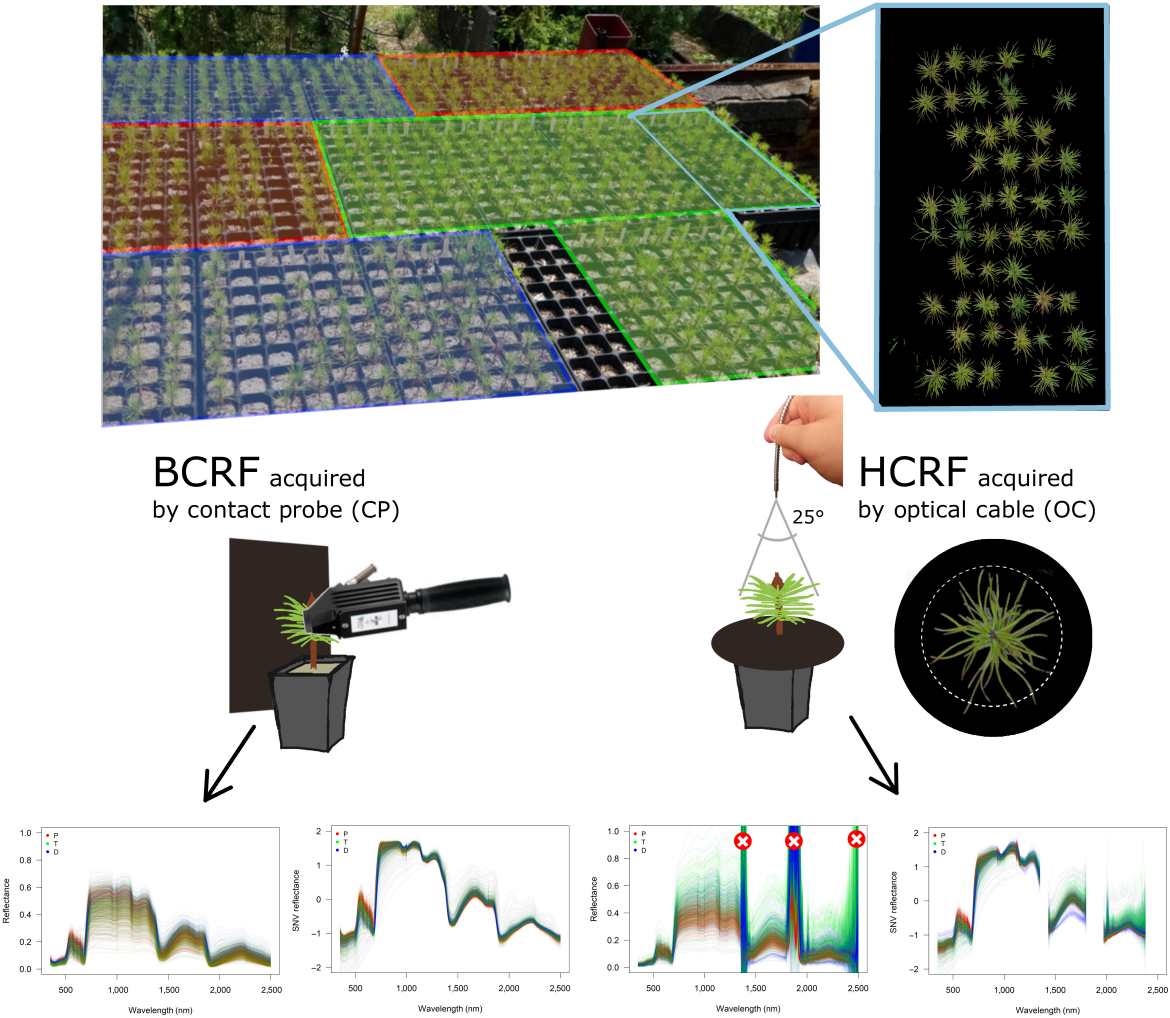
Experimental and technical design. Planters’ photo is a generic illustration of how the ecotypes were replicated; spectral reflectance measurements were done with a spectroradiometer equipped with a CP acquiring BCRF on the left and by OC acquiring HCRF on the right with an additional nadir image of the measured seedling. Data processing demonstrated on graphs of spectral reflectance curves with the *x* axis corresponding to wavelength (in nanometers) and the *y* axis corresponding to reflectance (%). Red circles with a white cross above maximum reflectance values in the third graph denote unreliable spectral regions that were removed. The second and fourth graphs illustrate the impact of SNV transformation.

The study was conducted using 1-year-old (their second growing season) Scots pine (*Pinus sylvestris* L.) seedlings originating from 3 seed orchards: Plasy (P), Třeboň (T) and Děčín (D) from the Czech Republic. Together, 1,788 seedlings were measured, specifically 746 seedlings from Plasy, 750 from Třeboň, and 292 from Děčín (Fig. 1). Seedlings were grown in planters with 60 individuals. In a single planter, there were always only seedlings from one of the seed orchards, and single half-sib families shared the same line of cells. The reflectance factor measurements were carried out between 7 and 10 September 2021, at the Sofronka arboretum in Pilsen, Czech Republic, where all seedlings were grown. It is worth mentioning that there were differences among seedling populations in terms of mortality rate during the first winter (2021 season) after germination (Table 1). The upland pine ecotype represented by the population from Děčín exhibited the highest mortality. In addition, seedlings from the Děčín population exhibited lower average height in comparison to seedlings from Plasy and Třeboň populations.

**Table 1.** Description of the seed origin (3 distinct local populations comprising lowland and upland ecotype) and a common garden site. Děčín plus trees were mostly selected in the elevation of 700 m above the sea level. Climatic variables for 2021 were adopted from https://www.inmeteo.cz/. The mean heights are reported with respective standard deviations.

	Seed orchards			Common garden
	Plasy	Třeboň	Děčín	Sofronka
**GPS coordinates**	49.9087617°N 13.4427394°E	49.0166106°N 14.8247583°E	50.8191364°N 14.1172131°E	49.789113°N 13.386193°E
**Elevation (m.a.s.l.)**	385	430	465	331
**Ecotype**	Lowland	Lowland	Upland	–
**Mean annual precipitation (mm)**	489	623	826	532
**Mean annual temperature (°C)**	11.1	11.1	8.9	8.5
**Mean annual sunshine hours (h)**	1,524.3	1,571.5	1,099.9	1,457.6
**Mean height of the progeny (mm)**	49.6 ± 9.6	51.2 ± 10.4	46.5 ± 10.4	–
**Mortality within the progeny (%)**	5.7	13.9	28.6	–

### Measurements of the reflectance factors

For the spectral data assessment of 1,788 Scots pine seedlings, we used ASD FieldSpec 4 Wide-Res portable spectroradiometer (Malvern Panalytical Ltd., United Kingdom). The reflectance factors were measured in a span of 350 to 2,500 nm with an integration time of 8 ms for CP and 34 ms for OC, respectively. Three sensors cover the spectroradiometer range, changing in 1,000 and 1,800 nm. The spectroradiometer was equipped with an OC and attachable CP (ASD Plant Probe, Malvern Panalytical Ltd., United Kingdom). In all seedlings, the leaf-level BCRF was measured using the CP, and the whole seedling HCRF was acquired by OC. The seedlings were measured in the same order by both methods to ensure an accurate match in the sequence of measured individuals within 4 consecutive days. In both spectra acquisition approaches, the reflectance factor of the white panel with approximately 99% reflectance was recorded every 15 min and/or after the change in incoming radiation and later used as a white reference. The reflectance of the white spectralon panel (SphereOptics, Herrsching am Ammersee, Germany) used to calibrate the raw data to reflectance reaches 98 to 99% reflectance up to 1,800 nm and further varies between 94% and 98% reflectance.

#### Leaf level BCRF measurement

BCRF refers to the ratio of the reflected flux collected through an OC coupled with a CP, where the incident flux is limited to a conical solid angle. The incident flux only includes light coming from a particular range of directions, in our case, an integrated light source (a krypton halogen bulb) inside the CP [53].

The contact measurement at the leaf level was not reliant on weather conditions. The needles were pushed to a nonreflective black surface in the entire measured spectral range to minimize the background spectral noise or radiation transmitted [46]. The needles were arranged to cover the field of view. Each measurement was recorded from 25 scans as protection against overheating.

#### Canopy level HCRF measurement

HCRF refers to the ratio of the reflected flux collected through an OC to the incident flux from the entire hemisphere. In this case, the incident flux encompasses light coming from all directions of ambient sky illumination. The reflected flux is measured within a specific conical solid angle by bare OC [53].

Canopy level reflectance factor measurements were collected under clear sky conditions, rendering stable incoming light. The white panel was repeatedly measured and used to calibrate to reflectance the closest in time, thus accounting for short-term variation under illumination conditions and the diurnal change in solar illumination. We applied black nonreflective paper stencils to the seedling bases to eliminate soil reflectance from the recorded signal. For each measurement, the OC was pointed at the nadir (i.e., positioned vertically) to the terminal bud of the seedling from approximately 5 cm in height. The OC’s field of view angle is 25°, so the entire seedling was included in the field of view, and the background was effectively minimized. Each measurement acquired recorded an average from 50 consecutive ASD scans.

### Data processing and statistical analysis

#### Data preprocessing

Because of prominent noise in HCRF spectra (Fig. 1), we removed the reflectance from the NIR (1,350 to 1,430 nm) and SWIR (1,800 to 1,970 nm and 2,380 to 2,500 nm) regions, strongly affected by atmospheric aerosols and scattering. Similar spectral range removal is a frequent practice when working with field spectra acquired under solar illumination [59,61]. Moreover, we transformed BCRF and HCRF for consecutive analysis by standard normal variate (SNV) [62]. To make all spectra comparable in terms of intensities, the SNV method subtracts each spectrum from its own mean, followed by a division by its own standard deviation.

#### Statistical analysis

For statistical analysis, native functions of the R software version 4.0.4 (R Core Team 2021) and the ASReml library for R version 4 [63] were used. A univariate linear mixed model was fitted to evaluate all traits of interest with the following terms:

$$\begin{array}{c} y=1\mu +X_{1}\beta_{x}+X_{2}\beta_{y}+X_{3}\beta_{\mathrm{xy}}+\mathrm{Zm}+e \end{array}$$

where ***y*** corresponds to the data vector; ***μ*** is the overall mean effect; ***β***_***x***_ is a fixed effect associated with sites of the origin; ***β***_***y***_ is a fixed effect of the measurement day; ***β***_***xy***_ is a vector of the fixed interaction between origin and measurement day; ***m*** is the maternal general combining ability, with ***m*** ~ N(**0**, σ^2^_m_***I***_***m***_); and ***e*** is the random vector of errors, with ***e*** ~ N(**0**, ***R***). The symbol **1** is used for a vector of ones, while ***X*** and ***Z*** designate incidence matrices associated with fixed and random effects, and ***I***_***m***_ provides an identity matrix of order m. The ***R*** matrix was evaluated assuming independent errors, i.e., ***R*** = σ^2^***I***_***m***_.

In ASReml software, we performed the Wald tests to compare ecotypes and populations within our study. Wald test is a pseudo-analysis of variance using incremental Wald statistics. The incremental Wald tests have an asymptotic χ^2^ distribution, with degrees of freedom given by the number of estimable effects [63]. For pairwise comparison, we based our predicted site means of seed origin on the above-described linear mixed model.

#### ML algorithms and predictions

Two ML algorithms, RF and SVM, were applied to predict the seedling population of origin based on the VSWIR representative reflectance dataset (spectral phenome). R software was used to implement RF and SVM. For RF, we used the randomForest function from the randomForest package (v 4.6-14) [64], with ntree = 1,000 and retaining all other default parameters. The importance of individual wavebands for the RF model was extracted using the importance function.

The SVM model was fit using the svm function from the e1071 package (v 1.7-3) [65] with default parameters. The raw and SNV-transformed spectra of BCRF and HCRF were used for ML prediction, and prediction results were compared.

For both OC and CP, data were either treated in whole or separated by the day of measurement to inspect whether the predictions within the light conditions of a single day would be more consistent (it concerns mostly the OC measurement). We used a cross-validation approach with 100 iterations, randomly dividing observations into training (90%) and testing (10%) subsets. We then reported the mean accuracy of predictions for different input data.

## Results

### Differentiation of population based on leaf and seedling-level reflectance factors BCRF and HCRF

**Fig. 2.**
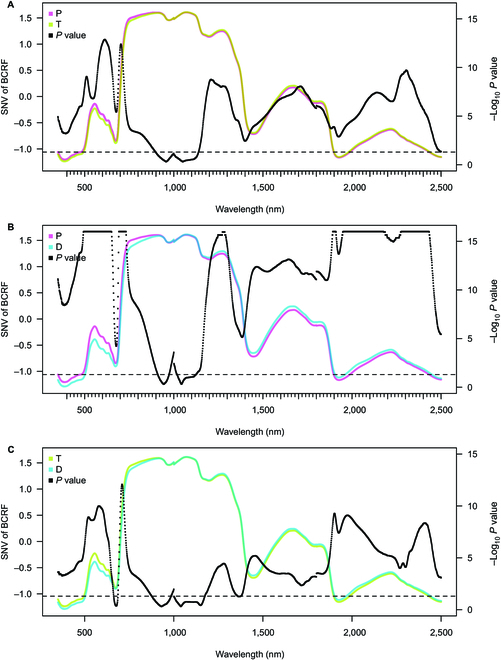
Average BCRF of seedlings from Plasy and Třeboň (A), Plasy and Děčín (B), and Třeboň and Děčín (C) measured by the CP. The reflectance spectral curve represents the average values from the individual sets of BCRF (SNV-transformed). The black points represent the negative decadic logarithmic *P* value of the pairwise comparison based on the linear model. *P* values of the pairwise comparison above the horizontal line represent a significant difference (*α* = 0.05) between the seedling populations from 2 seed orchards.

**Fig. 3.**
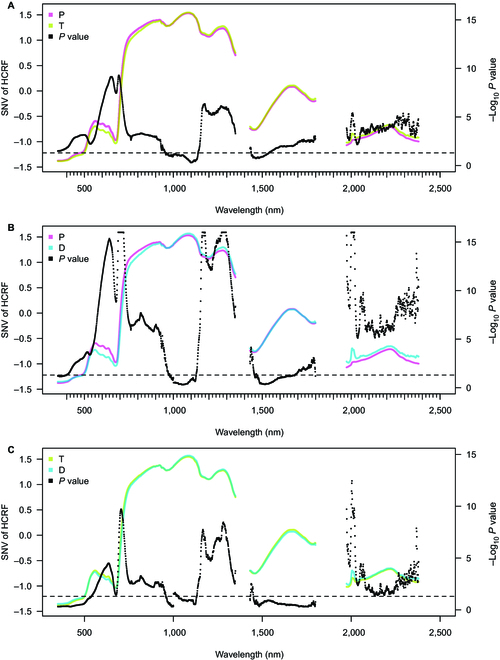
Average of HCRF of seedlings from Plasy and Třeboň (A), Plasy and Děčín (B), and Třeboň and Děčín (C) measured by the OC. The reflectance spectral curve represents the average values from the individual sets of HCRF (SNV-transformed). The black points represent the negative decadic logarithmic *P* value of the pairwise comparison based on the linear model. *P* values of pairwise comparison occurring above the horizontal line represent a significant difference (*α* = 0.05) between the seedling population from 2 different seed orchards. Because of prominent noise in HCRF spectra, we removed the reflectance from the NIR (1,350 to 1,430 nm) and SWIR (1,800 to 1,970 nm and 2,380 to 2,500 nm) regions.

The differences between the mean reflectance factors of seedlings according to their origin (P, T, and D populations) were tested by the mixed linear model for each wavelength of BCRF and HCRF separately, and the concluding *P* values are depicted in Figs. 2 and 3).

#### Leaf BCRF measured by the CP

The average BCRF of all seedlings populations was significantly different in pairwise comparison (*α* = 0.05) in the spectral regions measured by CP (Fig. 2). Differences in BCRF were the most pronounced within VIS and RE. In all 3 pairwise comparisons, a discrepancy occurred in the vicinity of 1,000 nm (sensor switch), where the *P* value exceeded the 0.05 significance threshold (in Fig. 2, it effectively dropped below the broken line, which indicates the threshold on the logarithmic scale). The pairwise comparison of the T and D populations exhibited smaller general differences in all spectral regions compared to the comparison between P and T (Fig. 2A) or P and D (Fig. 2B). Moreover, their difference was insignificant in a narrow region before RE (Fig. 2C).

#### HCRF of whole seedlings measured by the OC

In the case of the OC measuring method, the average of HCRF of all seedling populations differed significantly in pairwise comparisons (*α* = 0.05) across the VIS and RE and a substantial portion of the NIR and SWIR (Fig. 3). The reported *P* values exceeded the 0.05 significance threshold (*α* = 0.05), in the region near 1,000 nm (sensor switch) and 1,500 nm in all 3 comparisons. Pairwise comparisons of wavelengths in SWIR2 (beyond 2,000 nm) exhibited pronounced amplitude variation (Fig. 3A to C). Pairwise comparisons did not detect any significant differences between T and D in SWIR1 (1,430 to 1,970 nm), and these differences in SWIR2 approached the critical level (Fig. 3C).

#### Leaf and canopy, BCRF and HCRF, detection of different patterns among seedling populations

**Fig. 4.**
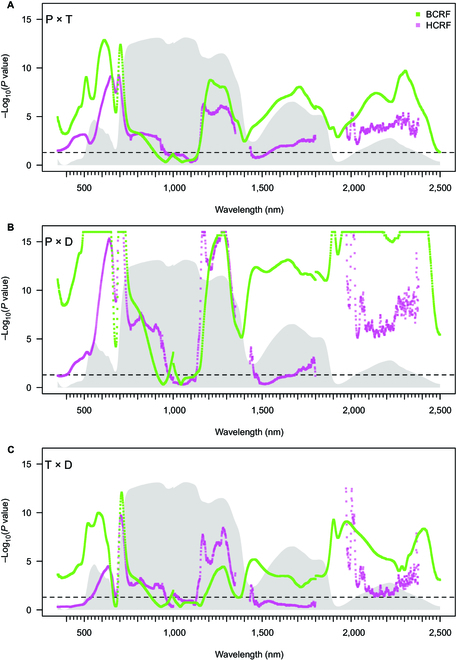
Two lines represent the negative logarithmic *P* value of pairwise comparisons of BCRF and HCRF of seedlings (SNV-transformed): Plasy and Třeboň (A), Plasy and Děčín (B), and Třeboň and Děčín (C) measured by CP (needles; CP = green; BCRF) and OC (whole seedling; OC = pink; HCRF). Gray shadow represents the average reflectance factor of the spectral curve. *P* values of pairwise comparison occurring above the horizontal dashed line represent a significant difference (*α* = 0.05) between the seedling population from 2 different seed orchards. Because of prominent noise in HCRF spectra, we removed the reflectance from the NIR (1,350 to 1,430 nm) and SWIR (1,800 to 1,970 nm and 2,380 to 2,500 nm) regions.

In general, *P* values of pairwise comparison of leaf and canopy BCRF and HCRF data of all 3 seedling populations maintained a similar trend at the VSWIR spectral range (350 to 2,500 nm). However, we encountered several discrepancies (Fig. 4) in *P* value patterns when visualizing these trends. *P* values of pairwise comparisons of BCRF are closer to zero than HCRF in a large part of the examined spectral interval. For the BCRF, the significance of the difference was most pronounced in the VIS, with a decrease in the red region (near 660 nm). RE is the second-highest peak in terms of significance for BCRF and HCRF. In the NIR, the significance of the comparison decreases toward the sensor switch (1,000 nm) and falls below significance for both BCRF and HCRF. In this region, the patterns are slightly different for BCRF and HCRF, but the relevance is questionable. In the spectral band between 1,100 and 1,400 nm, all pairwise comparisons of BCRF and HCRF based on the 3 populations exhibited significant differences. In the vicinity of 1,500 nm, the pairwise comparison of populations HCRF was insignificant compared to the BCRF. The main difference between the distribution of the *P* values between BCRF and HCRF in the population comparison occurred in the SWIR (1,430 to 1,970 nm). The *P* values of the BCRF (1,500 to 1,800 nm) are noticeably closer to zero, in contrast to the *P* values of HCRF. In the SWIR beyond the 1,970 nm, the *P* values of the BCRF were also closer to zero, with a lesser amplitude, than HCRF. Despite this observed difference, both HCRF and BCRF *P* values were significant within SWIR beyond the 1,970 nm.

### Using ML algorithms to distinguish seedling populations based on reflectance factors

We evaluated the ability of selected ML algorithms to separate the populations by comparing the predictions’ accuracy. We summarized the RF and SVM (Table 2) accuracies for raw and SNV standardized canopy HCRF and leaf BCRF. The accuracy (in percentage) indicates the probability with which the given method correctly assigns the seedling to the respective population of origin. We obtained the highest accuracy in the model based on raw whole seedling HCRF. The probability that HCRF of the seedling was assigned to the correct population reached 83% (RF) for day 1. The comparably worse results were obtained from the BCRF measured by the CP (54% to 68%, depending on measurement day and transformation). The maximum accuracy of BCRF prediction was 68% on day 2.

**Table 2.** The summarized accuracy values (%) represent the relative number of correctly assigned true origins based on 2 ML algorithms: RF and SVM. ML algorithms modeled BCRF raw values and HCRF raw values and their values normalized by SNV. Algorithms were compared on the first (day 1), the second day (day 2), and for both days together (both days).

	Day 1		Day 2		Both days	
	RF	SVM	RF	SVM	RF	SVM
	The summarized accuracy values (%)					
**HCRF**	73	64	66	67	65	67
**HCRF (SNV)**	83	81	71	72	71	73
**BCRF**	56	54	67	64	53	51
**BCRF (SNV)**	61	60	68	62	55	52

The SNV transformation led to an overall increase in accuracy no matter which data or ML algorithm was used. It is worth noting that prediction improvement was more visible in HCRF (6%) compared to BCRF (1% to 2%) when both days were included. This general trend was disrupted by day 1 of HCRF, where the difference made by SNV amounts to 10%. Similarly, day 1 of BCRF exhibited an increase in prediction accuracy (5%) after SNV transformation. The inconsistencies among measurement days will be further discussed.

#### Importance of specific spectral regions for RF separation of seedling populations

**Fig. 5.**
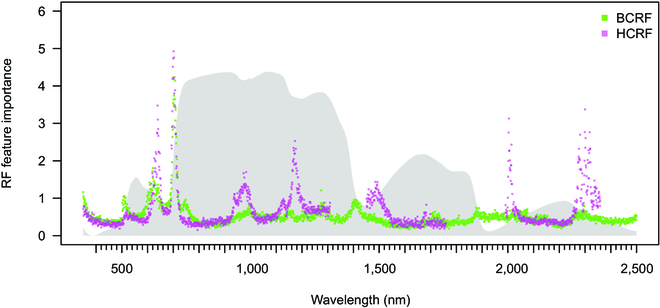
The points represent the prediction importance of each wavelength (350 to 2,500 nm) to the RF algorithm based on the BCRF (needles; CP = green; BCRF) and HCRF (whole seedling; OC = pink; HCRF). Gray shadow represents the average reflectance factor of the spectral curve. The spectra underwent the SNV transformation.

Another supporting evidence of why the BCRF and HCRF differ in their ability to predict seedling origin by RF was documented using the importance values of each wavelength for an RF model (Fig. 5). In both reflectance quantities, the importance showed similar peaks in VIS and RE. For BCRF, the important wavelengths for seedling population prediction were mostly limited to these wavelengths in VIS and RE. In contrast, HCRF showed several more spectral regions with high importance in NIR (900 to 1,200 nm and 1,100 to 1,200 nm) and SWIR (1,450 to 1,520 nm, 2,000 to 2,020 nm, and 2,280 to 2,380 nm). This difference in the number of spectral intervals with high importance for RF prediction between BCRF and HCRF is probably closely connected to the higher prediction accuracy of RF models based on HCRF data. The SNV transformation increased the prediction power of RF and SVM to a variable degree, which was also reflected in the patterns of importance values (Fig. S1).

## Discussion

In the last decade, hyperspectral phenotyping has been frequently applied in the evaluation of seedling susceptibility to drought [10], pollution [66], and early detection of various pathogen-induced diseases [32,67,68]. Spectral reflectance enables the efficient generation of multivariate datasets with high numbers of samples, including time series, ideal for discrimination of seedling conditions, using ML algorithms, which are an indispensable tool for hyperspectral data analysis [69,70]. There is also growing evidence that hyperspectral data contain information about inter- and intraspecific genetic variability [42,60] and the phylogenetic history of plant lineages [71]. In the present study, we demonstrate the ability of hyperspectral reflectance to distinguish individual populations of Scots pine seedlings. Two ways of measuring reflectance factors were compared—leaf/needle—BCRF by CP and whole seedling HCRF measurements by OC. The performance of ML classifiers (RF and SVM) was tested. Our goal was to investigate the benefits and limitations of BCRF and HCRF for the practical deployment of seedlings. We aimed to categorize them into similar groups to facilitate effective afforestation across the sites with varying ecological requirements without the risk of potential maladaptation.

### Differentiation of seedling populations based on leaf BCRF and seedling HCRF

The BCRF revealed a consistent, statistically significant (*α* = 0.05) difference among all studied populations across the measured spectral range (Fig. 2). The differences among populations were the greatest in the VIS part of the spectra in the regions related to photosynthetic pigments content. The significance peaked in the RE in all pairwise population comparisons. We interpret the maximum in BCRF interpopulation difference as evidence for the genetic background (local adaptation), supported by the peak of heritable variation in needle spectral reflectance in RE reported by Čepl et al. [72].

The studied populations differed significantly in BCRF also in NIR and SWIR regions. Considering the contact measurements of needle stacks flattened on a spectrally black background, their optical properties in NIR are mainly influenced by their internal anatomical structure. The volume and surface of intercellular spaces in mesophyll determine the multiple scattering and affect the leaf-level reflectance, as shown for red spruce and eastern hemlock [73]. The reason for BCRF detecting the population differences using the NIR may be based on the differences in their mesophyll structures, which should be addressed by needle anatomical analysis in the future. The dominant factor for leaf optical properties in the SWIR spectral region is the water content [74] and structural polymers. Schwanninger et al. [75] summarized spectral wavelengths, or spectral range, corresponding to lignin, cellulose, or other aromatics: 1,170, 1,143, 1,188 to 1,195 nm, 2,280 to 2,340 nm, and 2,330 nm [75], same as [33,76] and [77]. These specific wavelengths also exhibited significant differences among the populations in our study.

Similar to the pigment content variability, plant water management variation is connected to local adaptations under genetic control [78]. Therefore, the interpopulation differences in the SWIR reflectance region could be associated with genetically based differences in plant water management and structural compound accumulation. We have not conducted destructive analyses to measure the needle functional traits because the seedlings were used in a further phenotyping experiment.

The whole seedling HCRF acquired by the OC yielded similar pairwise differences between seedling populations to the leaf BCRF from CP. In the case of the average HCRF, all seedling populations differed significantly in pairwise comparisons (*α* = 0.05), similarly to BCRF, across the VSWIR. HCRF pairwise comparisons of seedling populations in SWIR1 and SWIR2 showed much less significance than BCRF. In longer SWIR2, the HCRF significance of pairwise comparisons (P × D and T × D) exhibited pronounced amplitude. However, the *P* value showed stronger fluctuation in SWIR2 compared to shorter wavelengths, probably due to the relatively low signal intensity as compared to the noise level.

The influence of water vapor still needs to be considered by excluding the noisiest NIR spectral bands [59,61]. As needle water content strongly alters the shape and magnitude of the reflectance curve in NIR and SWIR [74,79,80], the information about water content is lost if the spectral bands were removed from our dataset (especially near the 1,390 and 1,890 nm). It was shown that seedlings from different Scots pine provenances vary in their drought response and water management [81–83]. Thus, excluding noisy water absorption spectral regions may limit the distinction power among provenances. However, other informative water features could separate the ecotypes/populations in the 850- to 1,500-nm region. These limitations of field-measured HCRF may be a constraint for breeding application. Several growth traits (height, diameter, and crown length) in Norway spruce correlate strongly with needle reflectance in NIR and SWIR spectral regions, potentially useful in indirect selection [84].

To summarize, BCRF and HCRF were useful in determining the differences among the represented seedling populations in a common garden experiment. Except for the described differences between the 2 applied reflectance factor acquisition methods, both approaches share some similarities in the interpretation: mean BCRF and HCRF in the VIS spectral region of the D population were lower than T and P. The lower VIS mean reflectance of the D population could be attributed to the higher absorption. The D population might not be well adapted to our trial’s conditions corresponding to lowland climatic and environmental conditions, but its BCRF and HCRF did not indicate lower chlorophyll content. The D population’s lower average height and higher mortality compared to T and P populations could result from limited acclimation to trial conditions and provenance-specific growth rate (all 3 populations are significantly different in height, despite the relatively high variation within the populations).

In contrast, in the latter part of NIR and within SWIR, the BCRF and HCRF of Děčín seedlings were comparably higher, indicating variable water content that may be connected with the maladaptation. On a European scale, a trade-off was shown between height growth and stress tolerance in Scots pine (stress-resistant southern provenances with slower growth [82]). Thus, it is difficult for breeders to select elite offspring based on height at the juvenile stage, as Seidel and Menzel [81] demonstrated that taller and heavier Scots pine seedlings were more prone to drought-induced mortality. The main reason is the significantly different growth dynamics between ecotypes and generally low correlations between juvenile material and the rotation age of tree species [85].

### Impact of canopy biomass on reflectance factor measurement

Seedling biomass, morphology, and needle density or branching are additional features affecting reflectance factors measurements. On the one hand, when measuring BCRF, the needles were measured flattened against the black plate background; therefore, the effect of crown morphology was removed. On the other hand, HCRF was measured from above on the intact seedlings; thus, the leaf angle distribution of the needles and their density affected the reflectance. These influences are visible in provenance comparison and in ML predictions. Adult conifers have apparent differentiation into sun and shade needles according to the light exposure given by the vertical position within the canopy [86,87]. However, the seedling height was too small for needles to differentiate in this way.

### Prediction of the population origin based on the reflectance factor using ML

On the basis of the RF classification performance comparing the BCRF and the HCRF superior results, it can be concluded that the seedling morphology, which influences the HCRF data, contributes remarkably to classification accuracy. RF predictions resulted in higher accuracies from data with high variance and data that included information about seedling morphological differences. When these parameters are controlled or limited, as in the leaf-level CP data, the probability of correct origin prediction decreases. Therefore, the data obtained from whole seedlings by the OC (HCRF) yielded higher accuracy in predicting the 3 Scots pine populations.

Several machine learning algorithms have been previously used in distinguishing plant groups based on genetic variation at the subspecies level. Cavender-Bares et al. [42] applied partial least-squares discriminant analysis on BCRF to distinguish single oak species’ local populations. The same algorithm proved efficient in classifying hybrids between 2 cooccurring species of Dryas [60]. An SVM algorithm has been used for a similar case study to the present one, distinguishing *Tamarix* interspecific hybrids and pure species [59]. The classification performance of SVM was tested on both BCRF and HCRF acquired on *Tamarix* cuttings. However, the overall producers’ classification accuracies differed slightly (92% and 96% for BCRF and HCRF, respectively) [59]. The author also tested another ML algorithm, stochastic gradient boosting, with comparably high classification accuracies (91% and 93% for BCRF and HCRF, respectively) and claimed that stochastic gradient boosting is more suitable for their small sample numbers than the RF classifier.

The BCRF and HCRF differences in their prediction accuracy were described above and were further documented by the “importance values” (i.e., weighting factors) for each wavelength inferred from RF. Unfortunately, the information about wavelengths contributing significantly to the classification model is unavailable for every ML algorithm, including SVM. In RF classification using HCRF, the importance of each wavelength was evaluated and rated relatively highly across all spectral regions (including NIR and SWIR). In contrast, the importance peaks based on BCRF were limited to the VIS and RE regions. Čepl et al. [72] described the importance of the RE in the context of genetic variation, which complies with our findings.

Similarly, Cavender-Bares et al. [42] reported important wavelengths for classifying oak populations using partial least-squares discriminant analysis preferentially in the VIS and RE; the NIR and SWIR spectral regions also contributed to the model but less. This difference mentioned above between BCRF and HCRF may be connected to the variable prediction accuracy of RF models. Specifically, the importance peaks in the vicinity of 1,200 nm are unique for the HCRF data, and they might have been instrumental in the prediction of the D population (Fig. 5). Interestingly, this peak of importance coincides with the increasing *P* values in pairwise comparisons between D and P and between D and T populations, respectively.

The classification of the various tree populations at the subspecies level (populations of a pure species and their interspecific hybrids) based on reflectance has been recently reported [42,59,88,89]. However, predicting the specific population/provenance could be beneficial for selecting suitable individuals for breeding or reforestation. This classification should be done nondestructively concerning plant functional traits. The benefits of leaf-level spectroscopy are clearly the easy operation and relatively high throughput without sacrificing the measurement precision. Moreover, the proposed methods (OC/CP) do not rely on complex image segmentation and pixel classification connected with image-based spectroscopy. Although genomics in many tree species is still limited [90], genotyping using single-nucleotide polymorphisms has recently become more accessible for plant biologists and tree breeders. Currently, spatial genetics capitalizes on using spectral information about large and narrow scales [91]. Its ecology utilization has been predicted over a decade ago [92]. Genetic evaluation of hyperspectral data for breeding purposes was already efficiently combined with genotyping efforts [93,94]. Combining these 2 methods will accelerate the selection of seedlings with the required adaptation potential to the local environment [95].

### Limitations

Limitations inherent to each method may potentially affect the research findings and provide opportunities to improve in future investigations; thus, they are discussed in the following section.

Measuring BCRF by a CP equipped with an internal light source reduced the noise caused by ambient light conditions and atmospheric aerosol variation. The time spent measuring each sample should be adjusted to the plant material as the heat emitted from the light source could damage the younger leaves, especially when repeated measurements are needed. This drawback is particularly critical for studies dealing with induced drought stress. The other limitation of using CP in conifer seedlings is the risk of apical bud damage by the manipulation. The reason for slowing down the whole measurement process is the need to place a sufficient number of needles to cover the field of view, which will determine the quality and strength of the signal obtained. Considering the contact measurements of needle stacks flattened on a spectrally black background, their optical properties in NIR are mainly influenced by their internal anatomical structure. Therefore, the effect of crown morphology was removed. Measuring by CP is less dependent on the current conditions, its white reference is more stable, and measuring is comparable within the time.

HCRF measured by bare cable in the ambient illumination strongly depends on the current weather conditions. In contrast to CP-acquired BCRF, proximal OC measurements are less time-consuming when light conditions are stable. Thus, we can expect more consistent results in a shorter time on clear days and close to solar noon, reducing the variability given by changing light intensity and geometry. Aerosol absorption in the spectral ranges (especially near 1,390 and 1,890 nm) varied depending on the current conditions. These specific ranges were removed at the cost of losing water content information. Noncontact measuring also depends on the cable’s geometry, which the CP stabilizes. HCRF was measured from above the level of the seedlings; thus, the reflectance was affected by the angular distribution of needles and their density. Since the proximal measurement of whole seedlings depends on ambient light, the temporal changes under light conditions influence the predictions. To some extent, the effect of differences in light geometry and intensity is reduced by frequent white panel normalization and timing of the measurements only close to solar noon [59]. Measuring by OC also yields a weaker signal than measuring by CP.

Despite the limitations inherent to each reflectance acquisition method, they both proved viable for the manual HTP of Scots pine seedlings. We can hypothesize that the day-to-day differences in ML predictions were mainly due to the change in incoming illumination. However, the variation between days also affected CP-based ML prediction accuracies. In this case, it could most likely be attributed to the sampling effect—we measured a different subset of seedlings daily.

In the present study, we demonstrated that progeny populations of 3 different parent populations of Scots pine could be distinguished using their reflectance factors (BCRF and HCRF). The limitation of our study lies in the absence of supporting biophysical measurements of needle pigments, dry matter, water content, and morphological (needle density) seedling traits determined by traditional plant physiology approaches. The study was conducted on the seedlings designated for a drought resistance experiment; thus, the seedlings could not be sampled. However, we obtained the precise height of each seedling, and the populations’ progenies differ significantly in mean height. However, several similarly designed studies have shown that spectral reflectance may result in better classification than multiple leaf traits [42,89].

### Conclusion

The present study supports the emerging evidence that leaf and canopy reflectance spectra are useful to distinguish interpopulation variability and thus represent a valuable tool in high throughput plant phenotyping. Using reflectance factors and ML algorithms to detect various conifer seedling genotypes in a common garden or nursery experiment is a novel approach that could offer a vital tool for future effective nursery practices.

The present study was conducted on Scots pine seedlings, ecologically and economically important forest species with an extremely large spatial distribution and phenotype plasticity. Both reflectance factor measurement approaches proved viable in detecting differences among seedlings from 3 local populations (represented by 3 orchards), separating the mean BCRF and HCRF of the Děčín population that were well differentiated from Třeboň and Plasy in all cases. The classification models based on RF and SVM algorithms using HCRF that also included canopy structural information resulted in a higher probability of correct prediction of the respective population than BCRF. HCRF contactless measurement was faster and included morphological information, leading to higher prediction models’ accuracy. The proximal data obtained from the top of the seedlings offered over 80% accuracy in predicting 3 different Scots pine populations using RF and SVM algorithms.

Hyperspectral reflectance is gaining importance for breeding programs as measuring reflectance factors can be used for simple and nondestructive genetic evaluation. The present study’s take-home message is that manual hyperspectral phenotyping could provide an efficient tool in tree nurseries when a phenotyping unit is not affordable.

## Data Availability

The complete dataset is freely available upon request.

## Supplementary Materials

Supplementary 1 Fig. S1. Change of importance values after SNV transformation in BCRF and HCRF.
