# Supplementary material for: Making the Genotypic Variation Visible: Hyperspectral Phenotyping in Scots Pine Seedlings
Source: Plant Phenomics. 2023 Nov 14;5:0111. doi: 10.34133/plantphenomics.0111 (PMC10644830; doi:10.34133/plantphenomics.0111)
Supplement: Supplementary 1 — Fig. S1. Change of importance values after SNV transformation in BCRF and HCRF. [file plantphenomics.0111.f1.docx]

$y=1\mu+X_{1}\beta_{x}+X_{2}\beta_{y}+ X_{3} \beta_{xy}+Zm+e$

Supplementary Materials

Supplementary figures: Fig.S1


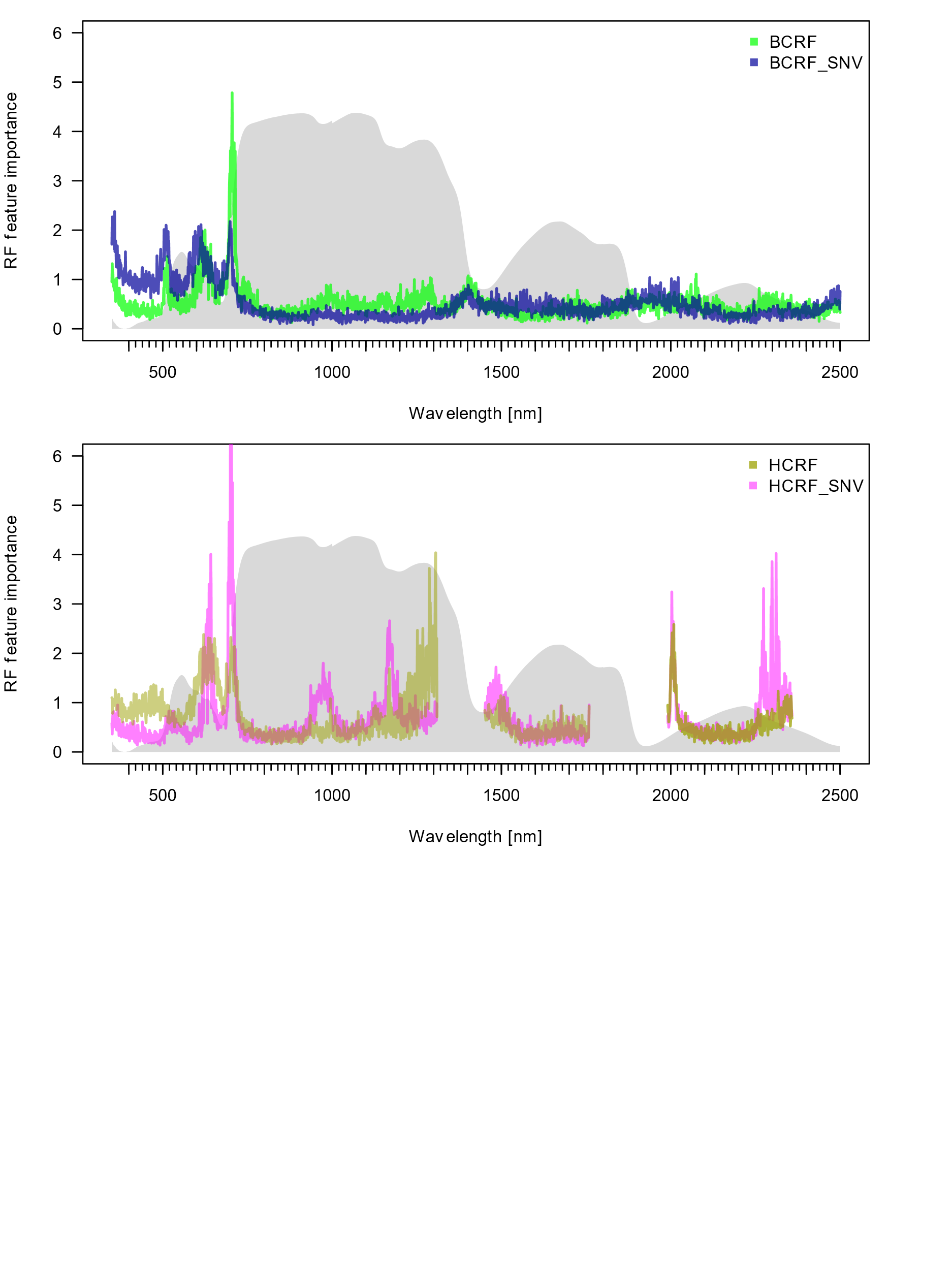


Fig.S1 – Change of importance values after SNV transformation in BCRF and HCRF. The points represent the prediction importance of each wavelength (350-2500 nm) to the Random Forest (RF) algorithm based on the BiConical Reflectance Factor and Hemispherical Conical Reflectance Factor with or without SNV transformation. Grey represents the average reflectance factor of the spectral curve.
